# Case Report: Looks can deceive: acute postoperative progressive renal dysfunction and hyperkalemia without significant hydronephrosis

**DOI:** 10.3389/fonc.2026.1685283

**Published:** 2026-01-30

**Authors:** Reza Lahiji, Ahmet Yildirim, William Luke, Lorenzo Storino Ramacciotti, Ernest A. Morton, Talia Avigail Helman, Jocelyn Nguyen, Valentina Grajales, Shreyas S. Joshi, Vikram M. Narayan, Behnam Nabavizadeh, Mohammad Hajiha, Reza Nabavizadeh, Peter J. Park, Kenneth Ogan, Mehmet A. Bilen, Viraj A. Master

**Affiliations:** 1 Department of Urology, Emory University School of Medicine, Atlanta, GA, United States; 2 Department of Hematology and Medical Oncology, Emory University School of Medicine, Atlanta, GA, United States; 3 Department of Urology, Weill Cornell Medicine, New York, NY, United States; 4 Department of Urology, Duke Cancer Center, Durham, NC, United States; 5 Department of Radiology and Imaging Sciences, Emory University School of Medicine, Atlanta, GA, United States; 6 Winship Cancer Institute of Emory University, Atlanta, GA, United States

**Keywords:** AKI, hydronephrosis, obstruction, RCC, ureter

## Abstract

**Introduction:**

Hydronephrosis is typically considered a hallmark radiographic finding of urinary tract obstruction, expected to develop within 7 days of onset. However, this timeline may not apply universally. We report a rare case of delayed-onset hydronephrosis in the context of borderline end-stage renal dysfunction following radical nephrectomy in a patient with metastatic renal cell carcinoma (RCC), emphasizing the limitations of imaging in the early diagnosis of obstruction.

**Case presentation:**

A 59-year-old male patient with metastatic renal cell carcinoma underwent right cytoreductive nephrectomy for an 8.5-cm renal mass with significant retroperitoneal lymphadenopathy and pulmonary metastases. Preoperative imaging showed mild left-sided hydronephrosis, and baseline renal function was preserved [estimated glomerular filtration rate (eGFR) 68 mL/min/1.73 m^2^]. Postoperatively, the patient immediately began experiencing a rapidly progressive decline in renal function, with eGFR falling to 15 mL/min/1.73 m^2^ and serum potassium rising to 7.0 mmol/L. Repeat imaging performed 8 days following initial decline was unremarkable, with findings consistent with prior scans. Subsequent nuclear medicine studies confirmed delayed perfusion and obstructive physiology in the solitary kidney. A nephrostomy tube was placed 15 days following the decline, leading to rapid improvement in renal function (eGFR 44 mL/min/1.73 m^2^ by POD 41).

**Discussion:**

This case illustrates the potential for clinically significant obstruction to occur in the absence of early hydronephrotic changes on imaging. We hypothesize that hyperacute progressive obstruction may lead to rapid intrarenal pressure stabilization, limiting capsular stretch and delaying radiographic findings. The absence of pain and significant radiographic hydronephrosis contributed to diagnostic delay.

**Conclusions:**

The absence of hydronephrosis on imaging cannot exclude obstruction, particularly in patients with a solitary functional kidney and high-risk features such as retroperitoneal malignancy. Early clinical deterioration should prompt a high index of suspicion and further diagnostic evaluation to prevent irreversible renal injury.

## Case report

**Figure 1:**
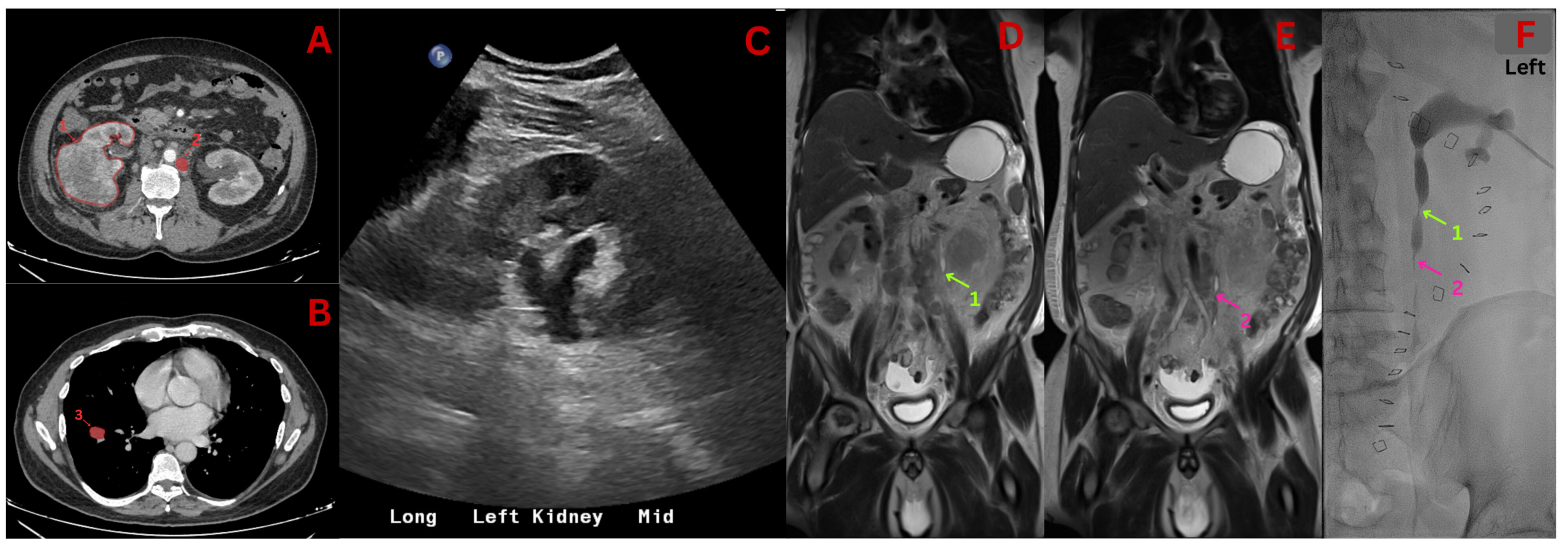
Radiographic imaging results. **(A)** CT scan revealing right sided renal mass (1) and an enlarged lymph node associated with periureteral stranding (2). **(B)** CT scan revealing lung metastasis (3). **(C)** Renal Ultrasound Scan revealing mild hydronephrosis. **(D, E)** Coronal MRI scans done near nephrostomy tube insertion, showing proximal ureteral obstruction (1 - green) and more distal obstruction (2 - pink). **(F)** Fluoroscopy revealing ureteric obstruction during nephrostomy tube insertion, correlated with arrows and numbers in figures **(D, E)**.

A 59-year-old male patient with a history of hypertension and remote smoking presented to an external outpatient urology clinic with genital swelling and nocturia for the previous 15 days. Following initial evaluation, the patient traveled overseas where he received a non-contrast CT scan significant for an 8.5-cm right renal mass, retroperitoneal lymphadenopathy, and bilateral pulmonary metastases (**Figures 1A, B**). Upon referral to our hospital, baseline laboratory results were notable for a creatinine level of 1.23 mg/dL, an estimated glomerular filtration rate (eGFR) of 68 mL/min/1.73 m2, and a potassium concentration of 4.5 mmol/L. Subsequent imaging detailed the underlying malignancy yet showed no signs of overt urinary tract obstruction bilaterally, apart from mild hydronephrosis in the left normal kidney with unremarkable renal function. The patient underwent a cytoreductive nephrectomy with plans for immunotherapy approximately 2 weeks following referral to our institution.

The patient underwent a right radical nephrectomy, with unresectable retroperitoneal adenopathy due to a remarkably intense desmoplastic reaction involving retroperitoneal nodes and great vessels. No significant immediate intraoperative complications were identified. Pathology revealed multifocal WHO/ISUP grade 4 clear cell renal cell carcinoma (ccRCC) with eosinophilic and papillary features, extensive necrosis, renal sinus and vein invasion, and adrenal metastasis (pT3a, M1). Subsequent Caris^®^ sequencing identified a pathogenic VHL mutation (*exon 3|p.e189fs*) and a BAP1 exon 6 frameshift (*p.G128fs*) mutation.

Postoperatively, the patient began developing rapidly progressive renal dysfunction, with an eGFR declining from 60 ml/min/1.73 m² on the day of surgery (DOS) to 28 ml/min/1.73 m² on discharge [postoperative day (POD) 3]. During a follow-up visit (POD 8), the patient was found to be hyperkalemic with a potassium level of 6.3 mmol/L and an eGFR of 25 mL/min/1.73m². The patient was treated for hyperkalemia, and a renal ultrasound was ordered. Ultrasound performed a week following initial deterioration demonstrated normal echogenicity and mild hydronephrosis (**Figure 1C**) in the remaining kidney, consistent with a previous preoperative imaging. Following intravenous therapy and observation, the patient had normal potassium levels and was discharged home.

Five days later (POD 13), follow-up blood work revealed a further decline in eGFR to 18 mL/min/1.73 m² and a potassium level of 7.0 mmol/L, necessitating emergent admission. MAG3 Lasix renogram performed the next day (POD 14) demonstrated delayed perfusion, poor cortical extraction, delayed peak time, and an unmeasurably long t½ (1).

Magnetic resonance imaging (MRI) performed just prior to nephrostomy placement (**Figures 1D, E**) revealed the obstruction and development of moderate hydronephrosis more than 2 weeks following initial deterioration. A nephrostomy tube was subsequently placed (POD 15), at which point the patient’s eGFR had declined to a nadir of 15 mL/min/1.73 m² and potassium remained elevated at 6 mmol/L (**Figure 1F**). The patient experienced an immediate improvement in renal function with a recovery of eGFR to 24 mL/min/1.73 m² and resolution of hyperkalemia 4 days later (POD 19). Following stabilization, the patient was initiated on ipilimumab -nivolumab immunotherapy (POD 19). The patient’s renal function has steadily improved, most recently measuring 44 mL/min/1.73 m² (POD 41).

**Figure 2:**
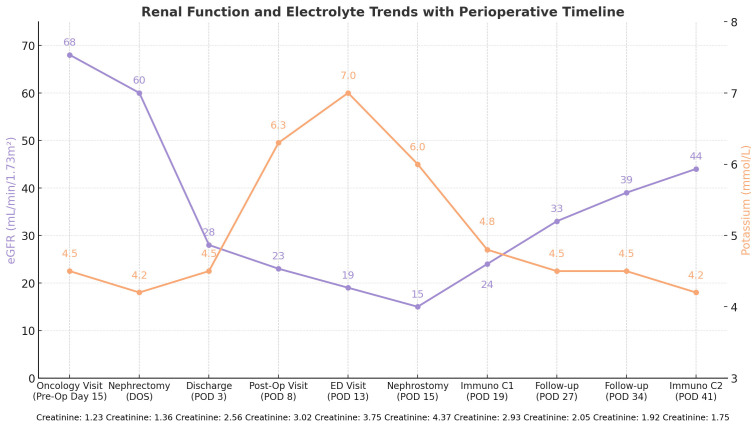
Timeline of events and corresponding lab value trends.

Of note, following nephrostomy tube insertion, the patient developed post-obstructive diuresis with between 6,500 and 8,500 mL of urine output per day for 3 days. Additionally, the patient’s weight decreased from 82.1 kg on discharge (POD 3) to 74.5 kg following nephrostomy insertion (POD 18), a 7.6-L decline in total body water. Throughout the clinical course, the patient remained consistently hypertensive with blood pressure fluctuations of approximately 150/90 mmHg. Currently, the patient is undergoing close surveillance regarding his renal function, response to immunotherapy, and further oncologic management. The overall timeline of events and the corresponding laboratory results are illustrated in **Figure 2**. Patient verbal and written consent was given for all materials included in this report.

## Discussion

This case illustrates a highly unusual presentation and postoperative course of metastatic RCC. To our knowledge, this is one of the few cases reported where significantly worsening hydronephrosis did not become apparent for more than 2 weeks following obstruction and significant renal dysfunction. This presentation contradicts current literature, citing a timeline of 7 days to the development of characteristic radiographic findings. Although initial preoperative imaging revealed only mild left-sided hydronephrosis and renal function appeared unremarkable, significant progressive obstruction-related impairments occurred following nephrectomy. A detailed understanding of the pathophysiologic underpinnings of ureteral obstruction is essential to contextualize the abrupt deterioration observed.

Glomerular-level pathological changes, which are hypothesized to ensue following obstruction of a solitary kidney, can be broadly categorized into three main phases (2, 3). The initial phase (phase 1; 1 -2 h following obstruction) describes afferent artery vasodilation to maintain glomerular filtration rate (GFR) (4, 6). During the second phase (phase 2; 2 -5 h following obstruction), efferent vasoconstriction and increased pelvic pressure on the interstitium result in reductions in renal blood flow (RBF) due to raised total renal resistance (4, 6). The final phase (phase 3; 5 -24+ h following obstruction) is mediated through vasoconstricting agents and involves a paradoxical reduction in ureteral pressure as both RBF and GFR decrease and urine production halts (4, 6). It is important to note that while complete ureteral obstruction for a short duration markedly reduces RBF and GFR, minimal anatomical changes in blood vessels, glomeruli, and tubules are produced (4).

**Figure 3:**
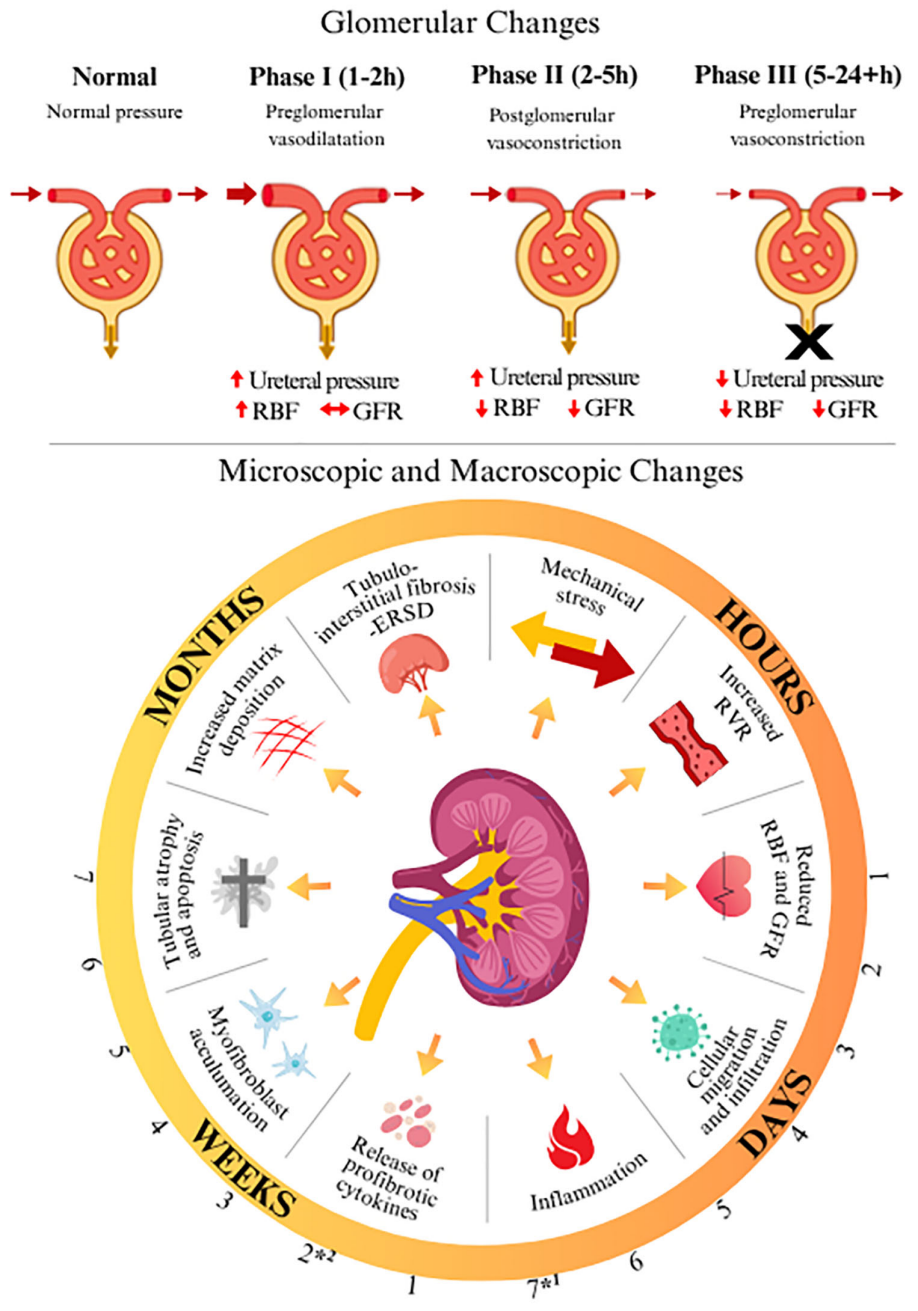
Timelapse demonstrating glomerular and functional consequences of ureteral obstruction (4–6). ^*1^ Radiographic hydronephrosis is said to become apparent in literature. ^*2^ Radiographic hydronephrosis became apparent in this patient (>2 weeks). Renal Blood Flow (RBF); Glomerular filtration rate (GFR); Renal vascular resistance (RVR); End-stage renal disease (ESRD).

Currently accepted gross pathological changes that occur in the kidney have been characterized in animal models, with parallels observed in humans (2, 7). After 42 h of obstruction, dilation of the collecting system and blunting of papillary tips ensue (7). At 7 days following obstruction, the renal parenchyma becomes edematous, with significant radiographic findings expected (2, 4, 7). A timeline of the hypothesized structural and function renal changes associated with obstruction detailed above is depicted in **Figure 3** (2, 4). While current literature cites a timeline of 42 h to 7 days to the development of hydronephrotic findings, in this case, hydronephrosis was not apparent for more than 2 weeks.

It is known that the absence of hydronephrosis on imaging does not exclude obstruction, necessitating a high index of clinical suspicion to prevent delayed diagnosis and irreversible injury. In this case, mild preoperative hydronephrosis associated with peri-lymphoureteral stranding may have represented a version of retroperitoneal fibrosis and/or urine extravasation, impairing urinary tract dilation. In this setting of chronic partial unilateral obstruction, decreased ipsilateral RBF is expected. It is possible that the postoperative inflammatory cascade may have contributed to the development of acute-on-chronic, complete urinary tract obstruction. We further hypothesize that in the context of rapidly progressive obstruction in the setting of relative afferent vasoconstriction, a sudden rise in renal pelvic pressure may have further decreased RBF, thus limiting urinary output and collecting system dilation. Prior studies have estimated that approximately 50% of patients presenting with urinary obstruction may present with standard renal sonography (8, 9).

This patient's postoperative pain was managed using multimodal analgesia involving acetaminophen, gabapentin, and methocarbamol. Interestingly, he did not require analgesia as an outpatient. Though pain is typically a reliable indicator of acute obstruction, some degree of mild, chronic urinary obstruction may have desensitized his collecting system and renal capsule to nociception (10). As such, worsening renal function in the context of significant retroperitoneal lymphadenopathy must be thoroughly investigated, especially if hydronephrosis of any severity is observed, to rule out post-renal origins of kidney injury (2, 7).

While the 7-day mark is frequently cited, some patients may take several weeks or longer to demonstrate typical imaging features, as was reflected in this case. Had our patient's obstruction not been relieved, obstructive sequelae could have resulted in chronic kidney disease, congestive cardiac failure, cardiac arrest, or death (11). Some of the contributing features that aided our diagnostic process and may be useful for other clinicians included the presence of a single functional kidney, retroperitoneal lymphadenopathy, significant desmoplastic reaction observed intraoperatively, and persistently worsened eGFR, creatinine, and potassium.

## Patient perspective

“I first went to the urology clinic because i had swelling in my penis and difficulty urinating. The doctor told me it might be prostate enlargement, like my father had, and gave me medication for it. But no imaging was done at that time, and later I wondered why they didn’t check further, like doing a sonography. Soon after, I traveled to india and had imaging there, which showed a mass on my kidney. I flew back to the u.s. within six days, and my children arranged my appointments at emory. After meeting my uro-oncologist and medical oncologist, they explained the diagnosis clearly and recommended surgery. I thought that removing the kidney, removing the source, would help stop the disease from spreading. The surgery was tough, but I trusted my doctors completely and followed their advice.

After the surgery, I felt very weak. My potassium was high, and my kidney function dropped. There was a blockage, and I ended up going to the emergency room. The next day, a tube was placed in my kidney, and I started feeling better. I was even able to walk every day again. looking back, I think doctors should be more aware of kidney function closely after surgery and act early before kidney function drops too far. If creatinine or potassium is rising and gfr is starting to fall, it’s better to act quickly and consider placing a tube early to prevent lasting damage. I also want other patients to be careful, sometimes there are no obvious symptoms of cancer. In my case, I had no pain, but I was losing weight quickly without trying. If you’re losing weight without dieting, it’s important to get checked. To other patients suffering from kidney cancer, I would say: don’t be scared, if you stay positive and fight with strength, you can defeat this disease.”

## Conclusions

This case highlights an uncommon postoperative complication of metastatic renal cell carcinoma, emphasizing the need for heightened clinical vigilance in similar scenarios. The time to development of typical hydronephrotic signs on radiography may vary significantly. Prompt recognition of obstruction and intervention has allowed for a significant renal recovery, prevented potentially fatal complications, and enabled initiation of systemic therapy.

## Data Availability

The data analyzed in this study are subject to the following licenses/restrictions: Patient identifiable information, deidentified information available upon reasonable request. Requests to access these datasets should be directed to ahmet.yildirim@emory.edu.
